# Circulating microRNAs as biomarkers for rituximab therapy, in neuromyelitis optica (NMO)

**DOI:** 10.1186/s12974-016-0648-x

**Published:** 2016-07-08

**Authors:** Adi Vaknin-Dembinsky, Hanna Charbit, Livnat Brill, Oded Abramsky, Devorah Gur-Wahnon, Iddo Z. Ben-Dov, Iris Lavon

**Affiliations:** Department of Neurology, Multiple Sclerosis Center and Laboratory of Neuroimmunology, and the Agnes-Ginges Center for Neurogenetics, Hadassah Hebrew University Medical Center, Ein–Karem, Jerusalem, 91120 Israel; Nephrology and Hypertension Services, Internal Medicine Wing, Hadassah Hebrew University Medical Center, Ein–Karem, Jerusalem, 91120 Israel; Department of Neurology, the Agnes-Ginges Center for Neurogenetics and Leslie and Michel Gaffin Center for Neuro-Oncology, Hadassah Hebrew University Medical Center, Ein–Karem, Jerusalem, 91120 Israel

**Keywords:** Brain-specific microRNAs, Circulating biomarkers, Rituximab, Aquaporin 4 (AQP4), Neuromyelitis optica (NMO)

## Abstract

**Background:**

Neuromyelitis optica (NMO) is a chronic autoimmune disease of the central nervous system (CNS). The main immunological feature of the disease is the presence of autoantibodies to Aquaporin 4 (AQP4+), identified in about 82 % of cases. Currently, there are no reliable biomarkers for monitoring treatment response in patients with NMO. In an effort to identify biomarkers, we analyzed microRNAs (miRNAs) in the blood of rituximab-treated NMO patients before and after therapy.

**Methods:**

Total RNA extracted from whole blood of nine rituximab-responsive NMO patients before and 6 months following treatment was subjected to small RNAseq analysis. The study included an additional group of seven untreated AQP4+ seropositive NMO patients and 15 healthy controls (HCs).

**Results:**

Fourteen miRNAs were up regulated and 32 were downregulated significantly in the blood of NMO patients following effective therapy with rituximab (all *p* < 0.05). Furthermore, we show that expression of 17 miRNAs was significantly higher and of 25 miRNAs was significantly lower in untreated NMO patients compared with HCs (all *p* < 0.05). Following rituximab treatment, the expression levels of 10 of the 17 miRNAs that show increased expression in NMO reverted to the levels seen in HCs. Six of these “normalized” miRNAs are known as brain-specific/enriched miRNAs.

**Conclusions:**

Specific miRNA signatures in whole blood of patients with NMO might serve as biomarkers for therapy response. Furthermore, monitoring the levels of brain-specific/enriched miRNAs in the blood might reflect the degree of disease activity in the CNS of inflammatory demyelinating disorders.

**Electronic supplementary material:**

The online version of this article (doi:10.1186/s12974-016-0648-x) contains supplementary material, which is available to authorized users.

## Background

Neuromyelitis optica (NMO) is a chronic inflammatory disease of the central nervous system (CNS), whose clinical features include mainly acute attacks of bilateral or rapidly sequential optic neuritis (eventually leading to visual loss in many patients), and transverse myelitis. Disease etiology of NMO is still unknown. However, it is known that the inflammatory processes in NMO are mediated by the humoral immune system and primarily target astrocytes [1, 2]. The most important evidence of this was the identification of the NMO-IgG antibody, anti-Aquaporin-4; NMO-IgG antibodies identify about 82 % of patients. At present, it is challenging to diagnose NMO patients that are negative for this marker from patients with other CNS demyelinating diseases, especially multiple sclerosis (MS).

Micro RNAs (miRNAs) function to modify the expression of target genes. miRNA-mediated gene regulation is critical during many biological processes including inflammation and neurodegeneration. Because each miRNA can regulate many target genes, the biological impact of dysregulation of a single miRNA can be considerable. Recent research has shown that miRNAs have potential as non-invasive biomarkers for the diagnosis and prognosis of disease as well as monitoring of treatment response [3]; miRNAs also represent promising novel targets for therapy [1]. Extracellular circulating miRNAs are remarkably stable [4]. Their stability is achieved via different mechanisms. They can be packaged in microparticles (exosomes, microvesicles, and apoptotic bodies) [5–7] or associated with RNA-binding proteins (Argonaute2 [Ago2]) or lipoprotein complexes (high-density lipoprotein [HDL]) [8] to prevent their degradation. The stability of miRNAs, coupled with advances in high-throughput technologies that provide the ability to perform a global analysis of miRNA expression profiling, has positioned miRNAs as ideal biomarker candidates. Recently, altered miRNA expression has been reported in several human autoimmune diseases, and miR-92a was suggested as a circulating biomarker for disease staging in MS. The single study on miRNAs in NMO revealed that several miRNAs have distinct expression levels in NMO patients compared with healthy controls (HCs) and patients with MS [9]. But, additional more comprehensive studies are needed to establish these miRNAs as diagnostic markers.

Currently, the most commonly used treatments in NMO are steroids, azathioprine, mycophenolate mofetil, and rituximab [10–13]. Rituximab is a chimeric anti-CD20 monoclonal antibody that depletes B cells. It is commonly used for treating B cell lymphoma and has been found to be effective in the treatment of autoimmune rheumatological and neurological conditions including NMO. Rituximab is currently considered the most effective therapy for preventing NMO exacerbations [14, 15]. No definite biomarker of response to therapy is presently available for monitoring patients with NMO. Current data relating to the correlation between AQP4-IgG titers and disease activity in the long-term course of NMO are inconsistent [16].

In this study, we aimed to identify miRNA biomarkers in the peripheral blood of patients with NMO and to monitor treatment response. We examined whether analysis of global miRNA expression in the blood of patients with NMO before and after treatment with rituximab could identify a distinct miRNA expression signature associated with therapy. This putative signature could serve as a predictor for response to therapy, with the goal of assisting in the individualized management of this disease.

## Methods

### Subjects

The patient cohort included 16 patients with NMO (14 females, two males; age 41 ± 14.3 years; disease duration, 5.3 ± 6.2 years; Expanded Disability Status Scale (EDSS), 4.8 ± 1.8), followed at the Hadassah MS Center. The relapse rate in the 2 years prior to the study was 1.25 ± 0.7. None of the enrolled patients had relapse or were treated with steroids for at least 30 days before their blood samples were drawn. 82.3 % of the NMO patients were positive for anti-AQP4. Brain magnetic resonance imaging (MRI) was normal or compatible with the diagnosis of NMO in all patients, and spinal MRIs revealed long extensive myelitis in 14/16 patients. None of the nine responders had relapses in the 2 years following rituximab therapy. Five patients with treatment failure had comparable clinical characteristics to the responders but experienced no decrease in relapse rate with rituximab therapy (four females, one male; age 46 ± 10.7 years; disease duration, 5.2 ± 4.0 years; EDSS, 5.2 ± 4.4; relapse rate in the 2 years prior to the study, 1.3 ± 0.44). The patients signed informed consent. Clinical data were collected from patients’ files. NMO patients were diagnosed according to the NMO diagnostic criteria [17]. An age- and sex-matched control group comprised 15 healthy individuals (10 females, 5 males; age 36.7 ± 9.4 years).

NMO-Ig seropositivity in the study cohort was determined using RSR ELISA assay in sera. The anti-AQP4 ELISA positive samples were also assessed using a cell-based assay (Euroimmun).

### Blood RNA isolation and miRNA quantification

A 4-ml blood sample was collected in EDTA tubes from each patient. Two hundred fifty microliters of whole blood was mixed with 750 μl of Tri-reagent BD (Sigma), supplemented with 20 μl of 5 N acetic acid, and frozen. RNA was extracted according to the manufacturer’s instructions. Gel electrophoresis confirmed the integrity of the RNA, and total RNA was quantified using Qubit 2.0 (Thermo Fisher Scientific Inc.).

### Small RNA sequencing

Total RNA was subjected to multiplexed small RNA cDNA library preparation. Library preparation entails ligation of barcoded 3′ adapters to 20 different samples, pooling of samples, ligation of a 5′ adapter, reverse transcription and polymerase chain reaction (PCR), as previously described [18], with modifications allowing multiplexing of several 20-sample libraries on a single HiSeq lane, namely, 40–100 small RNA libraries per lane. Libraries were sequenced on an Illumina HiSeq sequencer, and the information obtained was analyzed by an automated computer pipeline to decode and annotate small RNA reads [19]. Normalization of miRNA reads was performed by dividing each miRNA read frequency by the total number of miRNA sequence reads within the subsample, thereby correcting the variable sequencing depth in each subsample.

### Real-time PCR

Mature miRNAs were quantified using Perfecta® microRNA Assays (Quanta Biosciences), according to the manufacturer’s instructions. Real-time polymerase chain reaction (PCR) was performed on a StepOne real-time reverse transcription (RT)-PCR (Life Technologies) in triplicate for each sample. The fold changes of miRNAs were normalized to RNU6B, (ΔCT). The data is presented as 2^−ΔCT^. Primer sequences were designed based on the miRNA sequences obtained from the miRBase database (http://microrna.sanger.ac.uk/). Statistical significance was calculated using two-tailed *t* test.

### Statistical analysis

Statistical procedures on count data were based on DESeq2, a publically available R/Bioconductor package for analysis of differential expression in RNA sequencing experiments [20], as previously described [21].

## Results

### Identification of differentially expressed microRNAs before and after rituximab treatment and between HCs and patients with NMO

The main aim of our study was to identify miRNAs that are differentially expressed before and after rituximab treatment. Rituximab is the most effective therapy for patients with NMO, with a response rate of approximately 90 % [22, 23]. Blood samples were collected from nine NMO patients, classified as rituximab responders, prior to and at 6 months following therapy and from 15 age- and sex-matched HCs. To further analyze the differential expression between untreated NMOs and HCs, seven additional untreated seropositive NMO patients were also included in the study. Total RNA was extracted from the whole blood samples, and miRNA levels were quantified by deep sequencing analysis (RNAseq).

RNAseq results demonstrated that the expression levels of 32 miRNAs were decreased and of 14 miRNAs were increased significantly (*p* < 0.05) in the nine treated NMO patients following treatment with rituximab (Table 1). These nine samples from responders were further compared with those from five non-responders. Using real-time RT-PCR, we were able to show that miR-125 was significantly different for rituximab responders and non-responders (*p* = 0.03). The additional eight miRNAs that were analyzed were differently expressed in non-responders, but the differences were not statistically significant, most likely due to the small number of non-responders in our cohort. (Data from the three most significant miRNAs are presented in Additional file 1: Figure S1).

**Table 1:** Differential expression of miRNAs following rituximab therapy

Upregulated miRNAs			Downregulated miRNAs		
miRNA treated vs untreated	Fold change	*p* value	miRNA treated vs untreated	Fold change	*p* value
hsa-miR-16	1.99	0.00001	hsa-miR-125b (#^)	0.17	0.00001
hsa-miR-15a	2.65	0.00001	hsa-miR-760 (#^)	0.08	0.00005
hsa-miR-124	6.42	0.00012	hsa-miR-135a (#^)	0.18	0.00058
hsa-miR-26b	1.91	0.00044	hsa-miR-134 (#^)	0.1	0.00183
hsa-miR-7	3.8	0.00085	hsa-miR-138 (#^)	0.18	0.00515
hsa-miR-15b	1.79	0.00206	hsa-miR-423-3p (#)	0.4	0.00734
hsa-miR-451	1.85	0.00233	hsa-miR-100 (#)	0.31	0.00818
hsa-miR-454	2.09	0.00458	hsa-miR-125a	0.24	0.00925
hsa-miR-17	1.66	0.00735	hsa-miR-135b(#^)	0.18	0.01455
hsa-miR-144	2.13	0.01293	hsa-miR-203 (#)	0.2	0.01629
hsa-miR-1180	3.53	0.01837	hsa-miR-660 (#)	0.31	0.04582
hsa-miR-106a	1.71	0.01897	hsa-miR-30d	0.34	0.000002
hsa-miR-184	3.41	0.03243	hsa-miR-200c	0.09	0.00003
hsa-miR-374b	1.62	0.03371	hsa-miR-30a	0.18	0.00003
			hsa-miR-99a	0.1	0.00009
			hsa-miR-29a	0.25	0.00034
			hsa-miR-200b	0.1	0.00043
			hsa-miR-24	0.47	0.00076
			hsa-miR-30e	0.33	0.001
			hsa-miR-27b	0.38	0.00199
			hsa-let-7c	0.44	0.00711
			hsa-miR-375	0.14	0.00836
			hsa-miR-152	0.23	0.01717
			hsa-miR-148b	0.43	0.01862
			hsa-miR-361-5p	0.23	0.01937
			hsa-miR-221	0.46	0.02188
			hsa-miR-200a	0.21	0.02416
			hsa-miR-328	0.4	0.02717
			hsa-miR-92a	0.71	0.02719
			hsa-miR-27a	0.48	0.0307
			hsa-miR-181a	0.49	0.04338
			hsa-miR-143	0.38	0.04993

miRNAs with increased expression in NMO patients that were normalized following rituximab therapy are indicated by (#); brain-specific/enriched miRNAs are marked with (^)

**Fig. 1:**
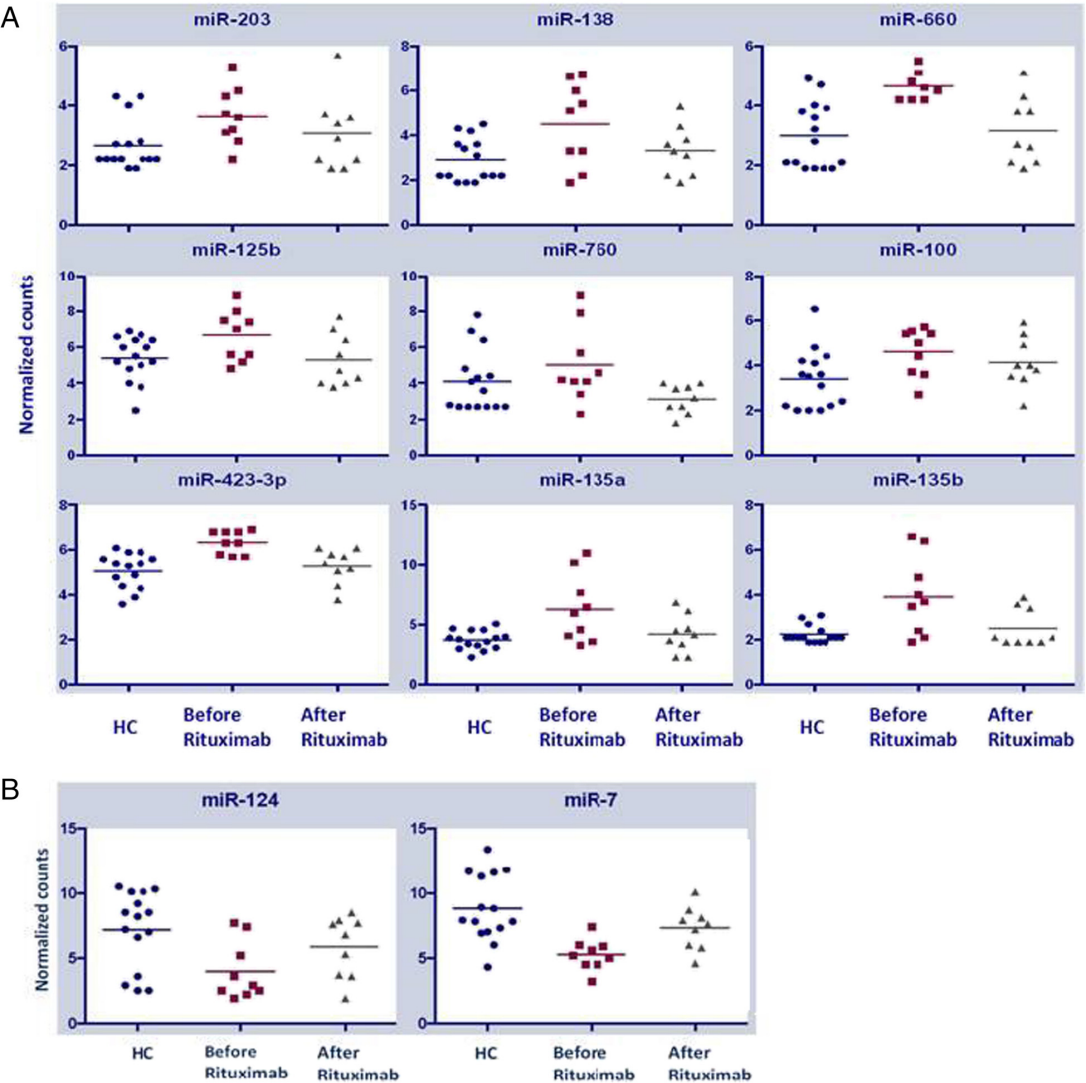
Differential miRNA expression between healthy controls and NMO patients before and following rituximab therapy. RNA was extracted from whole blood of 15 healthy controls and nine NMO patients before and after rituximab therapy, and miRNAs were quantified using deep sequencing. The results of nine of the 10 miRNAs which revert to normal levels following rituximab therapy are presented in the figure as normalized counts (logarithmic scale). Healthy controls, NMO patients before rituximab therapy and NMO patients after therapy are marked as *blue circles*, *dark red squares* , and *grey triangles*, respectively

Analysis of the differential expression between the 16 untreated NMO patients and 15 HCs demonstrated that expression levels were significantly lower for 25 miRNAs and significantly higher for 17 miRNAs in NMO patients compared with HCs (Table 2). Notably, the expression levels for 10 out of the 17 miRNAs with significantly increased expression in NMO patients reverted to the level of HCs following therapy with rituximab (Table 1, Fig. 1a). In addition, miR-7 and miR-124, which had lower expression in NMO patients, reverted to the level of HCs following therapy with rituximab (Table 1, Fig. 1b).

**Table 2:** Differential expression of miRNAs between untreated NMO patients and HCs

Upregulated miRNAs			Downregulated miRNAs		
miRNAs NMO vs HCs	Fold change	*p* value	miRNAs NMO vs HCs	Fold change	*p* value
hsa-miR-760 (#^)	10.6	0.00021	hsa-miR-7	0.19	0.00034
hsa-miR-423-3p (#)	1.6	0.0008	hsa-miR-140	0.4	0.00042
hsa-miR-660 (#)	6.1	0.00118	hsa-miR-22	0.57	0.00108
hsa-miR-135a (#^)	5.3	0.00202	hsa-miR-143	0.11	0.00109
hsa-miR-135b (#^)	8	0.00329	hsa-miR-150	0.35	0.00126
hsa-miR-1180	2.8	0.00562	hsa-let-7b	0.51	0.00221
hsa-miR-203 (#)	6.5	0.00862	hsa-miR-2110	0.14	0.00541
hsa-miR-18a	1.7	0.01101	hsa-miR-29a	0.28	0.00612
hsa-miR-20b	1.9	0.01591	hsa-miR-342	0.24	0.0103
hsa-miR-100 (#)	2.8	0.02	hsa-miR-10b	0.21	0.01041
hsa-miR-205	6.5	0.0228	hsa-miR-1227	0.12	0.01501
hsa-miR-134 (#^)	4.6	0.02493	hsa-miR-21	0.54	0.01947
hsa-miR-542	6.5	0.03172	hsa-miR-133b	0.16	0.01964
hsa-miR-125b (#^)	1.7	0.0413	hsa-miR-532-5p	0.33	0.02214
hsa-miR-551b	5.7	0.04761	hsa-miR-30e	0.47	0.03266
hsa-miR-454	2.1	0.04839	hsa-miR-133a	0.24	0.03284
hsa-miR-138 (#^)	3.5	0.04892	hsa-miR-139	0.23	0.04032
			hsa-miR-124	0.33	0.0424
			hsa-miR-130b	0.44	0.04353
			hsa-miR-378	0.48	0.0439
			hsa-miR-215	0.28	0.04642
			hsa-miR-411	0.19	0.04853
			hsa-miR-30a	0.32	0.04876
			hsa-miR-27a	0.46	0.04906
			hsa-miR-107	0.7	0.05042

miRNAs with increased expression in NMO patients that were normalized following rituximab therapy are indicated by (#); brain-specific/enriched miRNAs are marked with (^)

## Discussion

Our results demonstrate for the first time that a set of miRNAs with elevated expression in the blood of NMO patients revert to the levels of matched healthy controls following effective therapy with rituximab. The majority of these “normalized” miRNAs are known as brain-specific/enriched miRNAs. Following further validation, the results of this study might lead to the establishment of a diagnostic test to monitor treatment response in NMO.

The six brain-enriched miRNAs, miR-135a, miR-135b, miR-125b, miR-134, miR-138, and miR-760 [24–27], that revert to healthy control levels after treatment are expressed abundantly or predominantly in the brain. The higher quantity of these miRNAs in the circulation of patients with NMO might imply that they were shed to the circulation by degenerative brain cells, with a corresponding decrease in the amount of these miRNAs in the circulation observed following treatment, when the destructive inflammatory process diminishes. Therefore, monitoring the levels of brain-specific/enriched miRNAs in the blood might reflect the degree of disease activity in the CNS of demyelinating disorders.

miRNAs are highly stable elements that can be detected in body fluids as well as in different blood cells [28]. Their stability, along with the recent development of sensitive detection and quantification methods, has positioned miRNAs as great candidate biomarkers for diagnosis of specific diseases. Moreover, miRNAs might play a major role in initiating and/or maintaining autoimmunity processes [29, 30].

For many years, NMO was considered as a severe variant of MS [1]. Both diseases have autoimmune inflammatory lesions in the CNS. In MS, there is an abundance of data on miRNA expression in whole blood, peripheral blood mononuclear cells (PBMCs), plasma, cerebrospinal fluid (CSF), and peripheral blood T cells [31–34]. It is widely accepted that miRNAs are dysregulated in MS, but their use as biomarkers is still evolving [31]. In a recently published review on the available data regarding miRNAs in MS, Xinting et al. summarized that miR-15a, miR-19a, miR-22, miR-210, and miR-223 were upregulated in T-reg cells, plasma, blood cells, PBMCs, and brain white matter tissues from MS patients; miR-21, miR-142-3p, miR-146a, miR-146b, miR-155, and miR-326 were upregulated and miR-181c and miR-328 were downregulated in PBMCs and brain lesions; and miR-15a and miR-15b were downregulated in blood, peripheral T cells and B cells, or plasma samples from MS patients. None of the upregulated miRNAs were increased in our study; the distinct miRNA expression profiles provide further support for the concept that NMO has a distinct pathogenesis from MS.

In MS, there have been limited attempts to study the use of miRNAs in treated versus untreated patients, evaluating commonly used therapies. Waschbisch et al. studied five miRNAs (miR-20b, miR-142-3p, miR-146a, miR-155, and miR-326) by qPCR and found that there was significantly lower expression of miR-142-3p and miR-146a in glatiramer acetate (GA)-treated patients (no differences in expression were detected between untreated and IFN-β-treated patients) [35]. In an investigation of miRNA and mRNA expression in PBMCs of MS patients before and after IFN-β therapy, Hecker et al. found that IFN-β-responsive genes were upregulated in parallel to downregulation of miRNAs. Among the miRNAs, they identified altered expression among members of the miR-29 family [36]. Given the low clinical response rate of MS patients to IFN-β and GA therapy, the implications of these studies are unclear. For the more effective MS therapy, natalizumab, Sievers et al. found that 10 miRNAs, out of 1059 tested, were differentially expressed in B cells of natalizumab-treated patients versus untreated patients and Ingwersen et al. found that natalizumab therapy restored aberrant blood miRNA expression profiles in MS patients [37, 38]. The 10 most strongly upregulated miRNAs from MS lesions were expressed in astrocytes. This is noteworthy since in NMO the autoantibodies that are directed against AQP4 are expressed by astrocytes. This supports the rationale that miRNAs may contribute to pathogenesis of diseases in which astrocytes play a major role, like NMO.

In contrast to MS, in NMO, there is only one recent publication by Keller et al., which studied miRNAs in the blood of patients [9]. Their study included 11 patients with NMO, 60 patients with MS, and 43 HCs. They identified 141 differentially expressed miRNAs in NMO patients compared with HCs and 115 miRNAs in NMO versus MS patients. These results did not concur with the results from our study; not all the miRNAs that were found by Keller et al. were detected in our study and vice versa. The difference between the results of these studies may be partly attributed to the differences in patient cohorts. Our study included only untreated patients while most of the patients in the Keller et al. study were treated with immunosuppressive medications. Given the known influence of the immune system on miRNAs [39], it is conceivable that miRNA expression profiles would differ in immunosuppressant-treated and untreated patients. The use of different biostatistical method analyses and different RNAseq library preparation methods might also contribute to the different results [40]. Interestingly, one of the two RT-PCR-validated miRNAs identified by Keller et al. [9] was also highly significant in our cohort—miR-1180 (*p* = 0.005). Although our study demonstrated differentially expressed miRNAs in NMO patients and HCs, the main focus of our study was on differential miRNA expression after treatment. Using this analysis, we found a clear signature of miRNA expression that not only differed between NMO patients and HCs but also reverted to normal in response to rituximab therapy. Our study has its limitations and currently we cannot ascertain whether some of the above described changes in the miRNA levels following rituximab therapy are secondary to B cell depletion. It is noteworthy that the blood samples taken at the 6-month time-point were taken following analysis of positive CD19 cell detection in the blood, indicating that patients were not depleted of B cells. This supports the idea that the miRNA signature could represent a distinct phenomenon, rather than simply being a reflection of decreased B cell counts.

Rituximab is probably the most effective and commonly used therapy in NMO. Although most patients with NMO have detectable anti-AQP4 autoantibodies in the serum, the mechanism by which B cell-depleting activity mediates the beneficial effect is not clearly understood. Previous studies testing rituximab in NMO found a high response rate and most of the patients remained relapse free or experienced decreases in the annualized relapse rate (ARR) (60–100 % of disease-free patients in cohorts with 5–30 NMO patients) [22, 23].

Using 18 samples obtained from the nine responding patients in our study (two from each participant), we identified 46 miRNAs that significantly changed following rituximab therapy (*p* < 0.05) (Table 1). One of the significantly changed miRNA is miR-125. This miRNA has previously been described as a biomarker of rituximab therapy in B cell lymphoma and rheumatoid arthritis (RA) [41]. These findings give further validation to the authenticity of our analysis and suggest that the effect of rituximab on miR-125 levels may not be disease-specific.

Several attempts have been made to develop biomarkers in order to individualize rituximab therapy in NMO patients. CD19+ and CD27 + CD19 + B cell counts were studied by Kim et al. in NMO patients treated with rituximab [22, 42]. CD19 is expressed on B cells and CD27 on memory B cells. They reported that monitoring CD19- and CD27-positive B cell counts in the blood could help dosing the next rituximab administration. However, this assay of CD27-positive B cell suppression by rituximab is not in widespread clinical use. Recently, the same group found that, like in patients with non-Hodgkin lymphoma and RA [43], NMO patients carrying the V158F allele of the fragment c gamma receptor 3A (FCGR3A-F) have increased likelihood of incomplete B cell depletion and fivefold increase chance of relapse following rituximab treatment. This effect was more significant in patients that were not treated previously with methotrexate [43].

## Conclusions

In conclusion, we identified a miRNA signature in whole blood of patients with NMO. Following rituximab therapy, the expression levels of the majority of miRNAs (10/17) that have higher expression in patients with NMO versus HCs revert to normal levels following rituximab therapy. Our findings imply that this miRNA signature may serve as a biomarker for therapy response. Furthermore, six out of these “normalized” miRNAs are brain-enriched miRNAs. Monitoring the levels of brain-specific miRNAs in the blood of patients with inflammatory demyelinating disorders may reflect the degree of disease activity in the CNS.

## Abbreviations

AQP4, aquaporin 4; ARR, annualized relapse rate; CNS, central nervous system; CSF, cerebrospinal fluid; EDSS, expanded disability status scale; FCGR3A-F, fragment c gamma receptor 3A; GA, glatiramer acetate; HC, healthy controls; HDL, high-density lipoprotein; miRNAs, micro RNAs; MRI, magnetic resonance imaging; MS, multiple sclerosis; PBMC, peripheral blood mononuclear cells; PCR, polymerase chain reaction; RA, rheumatoid arthritis; RT, reverse transcription

## Additional files

Additional file 1: Figure S1. RNA was extracted from whole blood of nine responders and five non-responders NMO patients 6 months following Rituximab therapy and miRNAs were quantified using real-time RT PCR. The results of 3 most significant differentially expressed miRNAs are presented in the figure as 2^^Δ^ ct. Responders are marked in dark red and non-responders in grey. (DOCX 32 kb) Additional file 2: Table S1. miRNA normalized counts in patients with NMO. (XLSX 157 kb)
