# Supplementary figures and images for: Circulating microRNAs as biomarkers for rituximab therapy, in neuromyelitis optica (NMO)
Source: J Neuroinflammation. 2016 Jul 8;13:179. doi: 10.1186/s12974-016-0648-x (PMC4939003; doi:10.1186/s12974-016-0648-x)

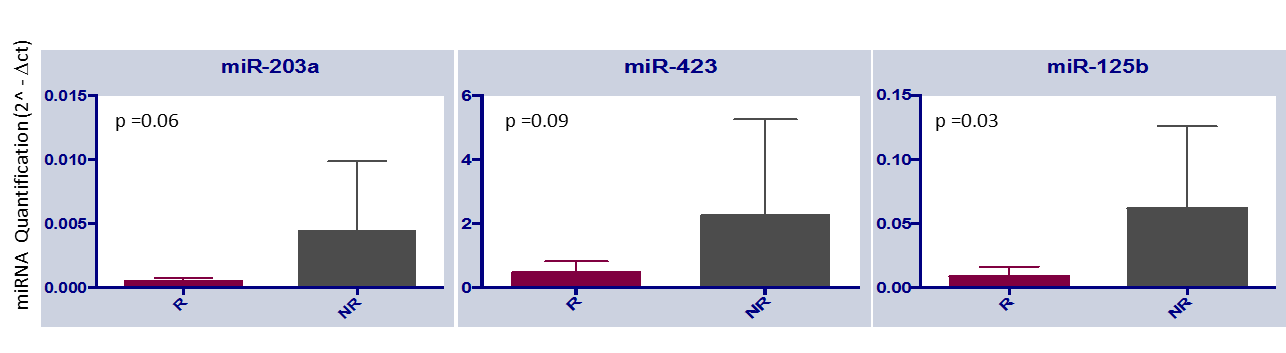
**Supplementary figure 1:**

Supplement: Additional file 1: Figure S1. — RNA was extracted from whole blood of nine responders and five non-responders NMO patients 6 months following Rituximab therapy and miRNAs were quantified using real-time RT PCR. The results of 3 most significant differentially expressed miRNAs are presented in the figure as 2^Δ ct. Responders are marked in dark red and non-responders in grey. (DOCX 32 kb) [file 12974_2016_648_MOESM1_ESM.docx]
